# Cryptic diversity, geographical endemism and allopolyploidy in NE Pacific seaweeds

**DOI:** 10.1186/s12862-017-0878-2

**Published:** 2017-01-23

**Authors:** João Neiva, Ester A. Serrão, Laura Anderson, Peter T. Raimondi, Neusa Martins, Licínia Gouveia, Cristina Paulino, Nelson C. Coelho, Kathy Ann Miller, Daniel C. Reed, Lydia B. Ladah, Gareth A. Pearson

**Affiliations:** 1 CCMAR- Centro de Ciências do Mar da Universidade do Algarve, Edifício 7, Gambelas, 8005-139 Faro Portugal; 2 Long Marine Laboratory, University of California, Santa Cruz, USA; 3 University Herbarium, University of California, Berkeley, USA; 4 Marine Science Institute, University of California, Santa Barbara, USA; 5 CICESE - Centro de Investigación Científica y de Educación Superior de Ensenada, Baja California, Mexico

**Keywords:** Allopolyploidy, Congeneric phylogeography, Cryptic species, Endemism, Fucaceae, *Hesperophycus*, Hybridization, Intertidal, NE Pacific, *Pelvetiopsis*

## Abstract

**Background:**

Molecular markers are revealing a much more diverse and evolutionarily complex picture of marine biodiversity than previously anticipated. Cryptic and/or endemic marine species are continually being found throughout the world oceans, predominantly in inconspicuous tropical groups but also in larger, canopy-forming taxa from well studied temperate regions. Interspecific hybridization has also been found to be prevalent in many marine groups, for instance within dense congeneric assemblages, with introgressive gene-flow being the most common outcome. Here, using a congeneric phylogeographic approach, we investigated two monotypic and geographically complementary sister genera of north-east Pacific intertidal seaweeds (*Hesperophycus* and *Pelvetiopsis*), for which preliminary molecular tests revealed unexpected conflicts consistent with unrecognized cryptic diversity and hybridization.

**Results:**

The three recovered mtDNA clades did not match a priori species delimitations. *H. californicus* was congruent, whereas widespread *P. limitata* encompassed two additional narrow-endemic species from California - *P. arborescens* (here genetically confirmed) and *P. hybrida* sp. nov. The congruence between the genotypic clusters and the mtDNA clades was absolute. Fixed heterozygosity was apparent in a high proportion of loci in *P. limitata* and *P. hybrida*, with genetic analyses showing that the latter was composed of both *H. californicus* and *P. arborescens* genomes. All four inferred species could be distinguished based on their general morphology.

**Conclusions:**

This study confirmed additional diversity and reticulation within NE Pacific *Hesperophycus*/*Pelvetiopsis*, including the validity of the much endangered, modern climatic relict *P. arborescens*, and the identification of a new, stable allopolyploid species (*P. hybrida*) with clearly discernable ancestry (♀ *H. californicus* x ♂ *P. arborescens*), morphology, and geographical distribution. Allopolyploid speciation is otherwise completely unknown in brown seaweeds, and its unique occurrence within this genus (*P. limitata* possibly representing a second example) remains enigmatic. The taxonomic separation of *Hesperophycus* and *Pelvetiopsis* is not supported and the genera should be synonymized; we retain only the latter. The transitional coastline between Point Conception and Monterey Bay represented a diversity hotspot for the genus and the likely sites of extraordinary evolutionary events of allopolyploid speciation at sympatric range contact zones. This study pinpoints how much diversity (and evolutionary processes) potentially remains undiscovered even on a conspicuous seaweed genus from the well-studied Californian intertidal shores let alone in other, less studied marine groups and regions/depths.

**Electronic supplementary material:**

The online version of this article (doi:10.1186/s12862-017-0878-2) contains supplementary material, which is available to authorized users.

## Background

Species diversity, endemism and structure are predicted to be lower in marine vs terrestrial assemblages because populations are less prone to diverge when dispersal potential is high and prominent barriers are absent [1–3]. Molecular markers, with their unprecedented power to delineate and describe biological relationships, show nevertheless a rather intricate and multi-layered picture of marine biodiversity. Many poor dispersers and/or habitat specialists often display extreme levels of cryptic phylogeographic structure across their wide distributions (e.g. [4, 5]), including divergent regional lineages of uncertain biological status (e.g. [6, 7]). True cryptic (and pseudo-cryptic, when morphologically distinguishable *a posteriori*) species are also commonly identified within morphologically conserved, hyper-diverse and/or poorly-studied taxonomic groups [8–10], and even within large, ecologically dominant organisms such as canopy-forming kelp [11, 12]. In the NE Atlantic, for instance, two new fucoids have been genetically identified (within existing taxa) in the past 10 years alone [13, 14].

Remarkably, the number of endemic (geographically restricted) cryptic species, often recovered within allegedly wide-ranging congeners, is also accumulating, particularly throughout tropical/subtropical archipelagos (e.g. [15–17]). Endemism may be a stable feature in the evolutionary history of some species, while for others just a transient state. For instance, nascent species are often spatially restricted, at least for some time [18]. Species may also endure climatically adverse periods in rather restricted “refugia”, before expanding their ranges again or becoming extinct [19–21]. In the terrestrial realm, many modern (interglacial) “climatic relicts” are concentrated in atypically cooler (or otherwise very heterogeneous) areas, such as southern mountain ranges [22]. Similar patterns may emerge, at least at the population level, in marine systems, for instance associated with cooler upwelling areas [23].

Modern phylogenetic analyses have also clarified marine species relationships and boundaries, often challenging traditional taxonomic classifications (e.g. [24–26]). Phylogenetic discordance and/or genomic admixture reveal that hybridization and gene introgression occur in a diverse range of marine taxa (reviewed in [27]). Perhaps unsurprisingly, hybridization has been found to be particularly frequent among sessile broadcast spawners (displaying external fertilization after release of the gametes in the water column) that form dense aggregations of closely related species, such as some corals [28] and canopy-forming seaweeds [29–31]. A range of sexual and clonal corals is suspected to be of actual hybrid origin [32, 33], but marine hybrid speciation, including via allopolyploidy (i.e., associated with chromosome multiplication), is otherwise very poorly documented. Evidence for ancient polyploidization events and for modern (allo)polyploid taxa has been accumulating across much of the tree of life [34–38], and its incidence and evolutionary significance may be more general than previously appreciated.

*Hesperophycus californicus* and *Pelvetiopsis limitata* are the extant members of two monotypic genera of canopy-forming fucoid seaweeds (Fucaceae, Heterokontophyta) endemic to the temperate rocky-shores of the north-east Pacific. Both occur in mid/high intertidal assemblages, although with complementary biogeographical distributions. Warm-temperate *H. californicus* is present from Punta Eugenia (Mexico) to Monterey Bay (California, USA), whereas cold-temperate *P. limitata* occurs from Point Conception (California) to Vancouver Island (British Columbia, CAN). A third putative species, *P. arborescens*, was described from just south of Monterey [39], but has never enjoyed much practical recognition and use. Collectively, these species/genera form a monophyletic lineage that is sister to *Fucus* [40], a much more speciose genus (8+ species) with a centre of diversity in the north Atlantic. The genus *Fucus* has been the focus of much physiological, ecological, and phylogeographical research [41–43], but reconstructing its evolutionary history has long been complicated by poor morphological discrimination, low marker resolution, incomplete lineage sorting, and hybridization/introgression [14, 24, 31, 44, 45]. *Hesperophycus*-*Pelvetiopsis* have received considerably less attention, but preliminary tests for newly developed molecular markers immediately revealed multiple taxonomic and phylogenetic conflicts consistent with cryptic diversity and reticulate evolution.

This study aimed to identify all genetic entities present within this complex, and to describe their phylogenetic relationships, morphological differences and biogeographical ranges. We reveal a single genus (we retain *Pelvetiopsis*) with four species, adding to this complex two narrow-endemic, pseudo-cryptic species from California (*P. arborescens* and *P. hybrida* sp.nov.), the latter of recent allopolyploid origin, a process so far unknown in brown seaweeds.

## Methods

### Sampling, DNA isolation and amplification, genotyping and sequencing

A comprehensive panel of populations of *Pelvetiopsis limitata sensu lato* (i.e., including putative *P. arborescens*) and *Hesperophycus californicus* was sampled along the entire ranges of these species in the NE Pacific. Preliminary results showed that *P. arborescens* and a fourth entity were genetically distinct, so additional populations (mostly from the Californian counties of Monterey and San Luis Obispo) were sampled to include all the morphological and genetic diversity present in the complex. Whole individuals were also sampled for descriptions of the general morphology of each recovered genetic entity. These were collected at the sites where they had been genetically identified and morphometric measurements, photographs and herbarium pressings were made. In total, 36 population samples containing 16 individuals (*ca*. 576 individuals) from 30 sites spanning Punta Baja (Baja California, Mexico) to Cape Meares (N Oregon, USA) were analysed (Additional file 1). Samples of apical tissue were collected every meter along 30 m, or haphazardly along 25–100 m, depending on the local spatial patchiness at each site, and were preserved dehydrated in silica-gel crystals until DNA extraction. Genomic DNA was extracted from *ca.* 8 mg dehydrated tissue using the Nucleospin® 96 Plant II kit (Macherey-Nagel Duren, Germany), and diluted 1:100.

All individuals were screened for the 23S/trnK mitochondrial intergenic spacer (mtIGS), as this marker was previously shown to provide good inter- and intra-specific resolution in the sister genus *Fucus*, and some propensity to cross species barriers [29, 31, 44]. The ‘universal’ fucoid/kelp primers of [46] were used to amplify a large 1.5 kb fragment spanning the mtIGS in a few individuals. New primers were designed in conserved genic flanking regions from these sequences to specifically target the mtIGS of *Hesperophycus*-*Pelvetiopsis* (see Additional file 2 for primer sequences and amplification details).

A geographically diverse panel of individuals representing all genetic entities inferred with the mtIGS was used to assess cross-amplification and polymorphism of 40 microsatellite loci developed *de novo* for *P. limitata*. These were identified *in silico* by screening with MsatCommander [47] a genomic DNA library (Biocant, Cantanhede, Portugal; http://www.biocant.pt/) using shotgun 454 pyrosequencing. From these, 13 loci were selected to produce multi-locus genotypes for all *Hesperophycus-Pelvetiopsis* individuals (see Additional file 2 for primer sequences and amplification conditions).

Amplified fragments were analyzed in an ABI PRISM 3130xl automated capillary sequencer (Applied Biosystems) at CCMAR, Portugal. Amplicons were pre-cleaned with ExoSap (Fermentas, Waltham, MA, USA) and sequences were aligned, proofread, and edited in GENEIOUS 4.8 (Biomatters; http://www.geneious.com). Microsatellite alleles were manually scored in STRand [48] using the GeneScan™ 500 LIZ™ size standard (Applied Biosystems).

### Data analysis

Haplotype/sequence networks were constructed in Network 4 (www.fluxus-engineering.com) using the Median-Joining (MJ) algorithm [49]. Phylogenetic relationships were reconstructed using Bayesian and Maximum Likelihood (ML) inference methods. Nucleotide substitution models (using three substitution schemes) were compared with jModelTest 2 [50] and the best-fit model selected based on the Akaike information criterion. Bayesian analyses were performed using MrBayes 3 [51]. Two parallel Metropolis-coupled Markov chain Monte Carlo searches, each with four chains (3 ‘heated’), were run for 10^6^ generations, sampling trees and parameters every 10^2^ generations. The number of substitution rates, among-site rate variation, and base frequency priors were set according to the substitution model selected, leaving the remaining options as default. Average standard deviation of split frequencies between runs and cold chains Log-likelihood stationarity were inspected to assess inter-run convergence and run length sufficiency, respectively. Based on the latter, 10^5^ generations (1000 trees) were discarded as burn-in. The remaining 18 000 trees sampled were used to produce 50% majority-rule consensus trees and to calculate branch posterior probabilities. Maximum likelihood analyses were performed with PhyML 3 [52] using the ATGC bioinformatics platform (http://www.atgc-montpellier.fr/phyml/). Nodal support was calculated using 1000 bootstraps. Trees were rooted with *Fucus* spp*.* Inter-specific divergence within *Pelvetiopsis* and within the two major lineages of *Fucus* was compared by estimating Kimura’s two parameter (K2P) distances between ancestral haplotypes defining each recovered species.

Summary statistics of the microsatellite genetic diversity, including microsatellite allele frequencies, mean allelic richness (*A*), Nei’s gene diversity (*H*_E_), observed heterozygosity (*H*_O_), and inbreeding coefficients (*F*_IS_) were calculated with Genetix 4 [53] for all populations. The number and delimitation of multilocus genotypic clusters and its congruence with mtIGS phylogroups was visually inspected with a factorial correspondence analysis (FCA) implemented in Genetix, and with a Bayesian, model-based genetic admixture analysis implemented in STRUCTURE 2 [54, 55]. In the latter, individuals were pooled into one dataset for analyses, without a priori population assignments. Each number of assumed genetic clusters (K, set sequentially from 2 to 4) was run five times using a burn-in of 200,000 iterations and a run-length of 1,000,000 iterations.

## Results

**Fig. 1:**
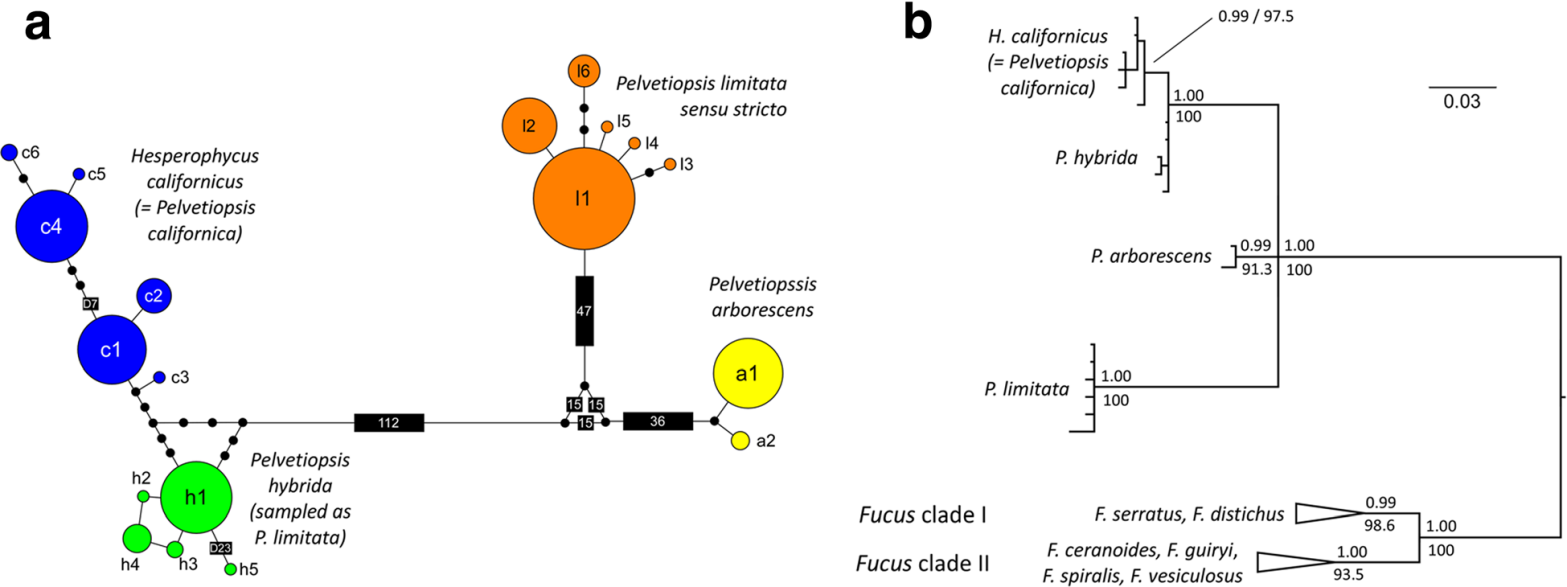
Genealogical relationships of *Pelvetiopsis* spp. based on mtIGS data. **a** MtIGS haplotype network; the *dots* represent inferred, unsampled haplotypes and the *small rectangles* represent inferred indels. **b** Reconstructed 50% majority-rule consensus phylogenetic tree. Numbers above and below the branches are Bayesian posterior probabilities (>0.90) and maximum likelihood bootstrap support values (>70), respectively. For clarity, outgroup (*Fucus*) branches were collapsed (*tip triangles*), with the horizontal length of the triangle representing the distance from the branches’ common node to the tip of the longest branch

A total of 19 mtIGS haplotypes (plus outgroups) were recovered. The mtIGS recovered three main clades that partially conflicted with a priori species delimitations. *H. californicus* was congruent, whereas three well resolved phylogroups were apparent within *P. limitata sensu lato* (Fig. 1). These included (see results below and Discussion): 1) *P. limitata sensu stricto* (the most widespread, hereafter simply referred to as *P. limitata*), 2) *P. arborescens*, and 3) a new, undescribed cryptic species, *P. hybrida* sp. nov., genealogically closer to *H. californicus*. MtDNA clades were well resolved (i.e. had high statistical support) but resolution was insufficient to establish the sequence of cladogenesis (lineage splitting, Fig. 1b). Mean sequence distances between *Hesperophycus-Pelvetiopsis* clades ranged between 0.057 (*P. arborescens* vs *H. californicus*) and 0.122 (*H. californicus* vs *P. limitata*), the same magnitude of that found between the two major lineages of *Fucus* (0.086, Table 1). Haplotype diversity within inferred entities was rather low (2–6 haplotypes, Fig. 1a), with 23 populations (~64%) fixed for a single haplotype (data not shown).

**Table 1:** MtIGS divergence in *Fucus* and *Pelvetiopsis*

	Fucus		Pelvetiopsis		
	distichus et al.	spiralis et al.	californica	arborescens	limitata
distichus et al.	0.029 ± 0.012 (3)	**0.086 ± 0.008 (15)**	-	-	-
spiralis et al.		0.023 ± 0.017 (10)	-	-	-
californica			0.010 ± 0.006 (3)	**0.057 ± 0.004 (3)**	**0.122 ± 0.006 (3)**
arborescens				(0)	0.075 (1)
limitata					(0)

Kimura’s two-parameter (K2P) sequence distances (mean ± sd) within and between (bold) major mtIGS clades of *Fucus* (each defined by one case species, for complete list see Cánovas et al. 2011 [40]) and *Pelvetiopsis*. The number of sequence comparisons is shown in brackets. *P. californica* is a synonym of *Hesperophycus californicus*

**Fig. 2:**
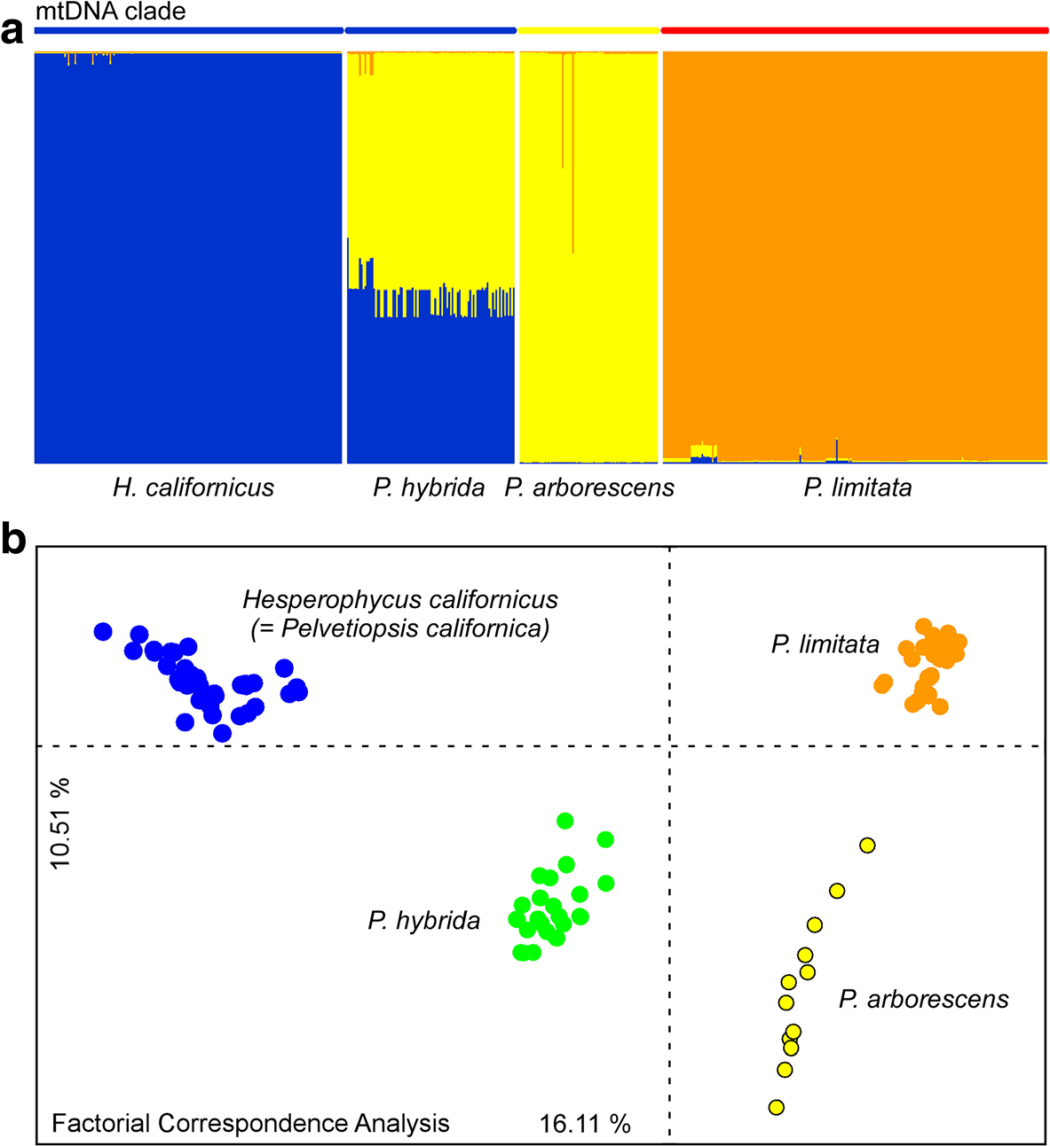
Genotypic entities within *Pelvetiopsis* as assessed with microsatellite loci. **a** Structure plot for K = 3, showing inferred taxonomic entities at the bottom and mtDNA clades at the top. **b** FCA plot, with individuals labelled according to inferred genetic entities. Note the congruence between sequence and typing data and between different analyses

Patterns of microsatellite cross-amplification and polymorphism varied markedly between loci, with four (Pl310, Pl51, Pl54, Pl311) failing to produce clear products in at least one species, and four (Pl32, Pl36, Pl51, Pl53) revealing inter-specific differences only (Additional file 3). Populations of *H. californicus* and *P. arborescens* typically exhibited homozygote excess (Additional file 1). In contrast, fixed heterozygosity was apparent in a high proportion of loci that successfully amplified in *P. limitata* (6/13) and *P. hybrida* (8/10) (Additional file 3), resulting in extremely high (and significant) heterozygote excess (-1.0 < *F*_IS_ < -0.6) in all their populations (Additional file 1). The occurrence of 3 alleles at a single locus was very rarely observed. In *P. hybrida*, each allele in heterozygote loci was identical or similar in size to either *P. arborescens* or *H. californicus*, although some peak imbalance (allele drop-out) was noticeable at loci Pl41 and Pl27 (Additional file 3). Homozygosity was only observed when *P. arborescens* or *H. californicus* produced similar-sized alleles (Pl53), or when amplification failed in one of them (Pl51). In *P. limitata*, alleles were shared with *H. californicus* or *P. arborescens*, or were species-specific. The congruence between the recovered genotypic clusters and the mtDNA clades was absolute (Fig. 2). As expected given its fixed heterozygosity and allele sizes, both Structure (K = 3; Fig. 2a) and FCA (Fig. 2b) analyses indicated that *P. hybrida* was genetically intermediate (hybrid) between *H. californicus* and *P. arborescens*. Contrastingly, allele states didn’t reveal any obvious genealogic relationship between *P. limitata* and its extant congeners.

**Fig. 3:**
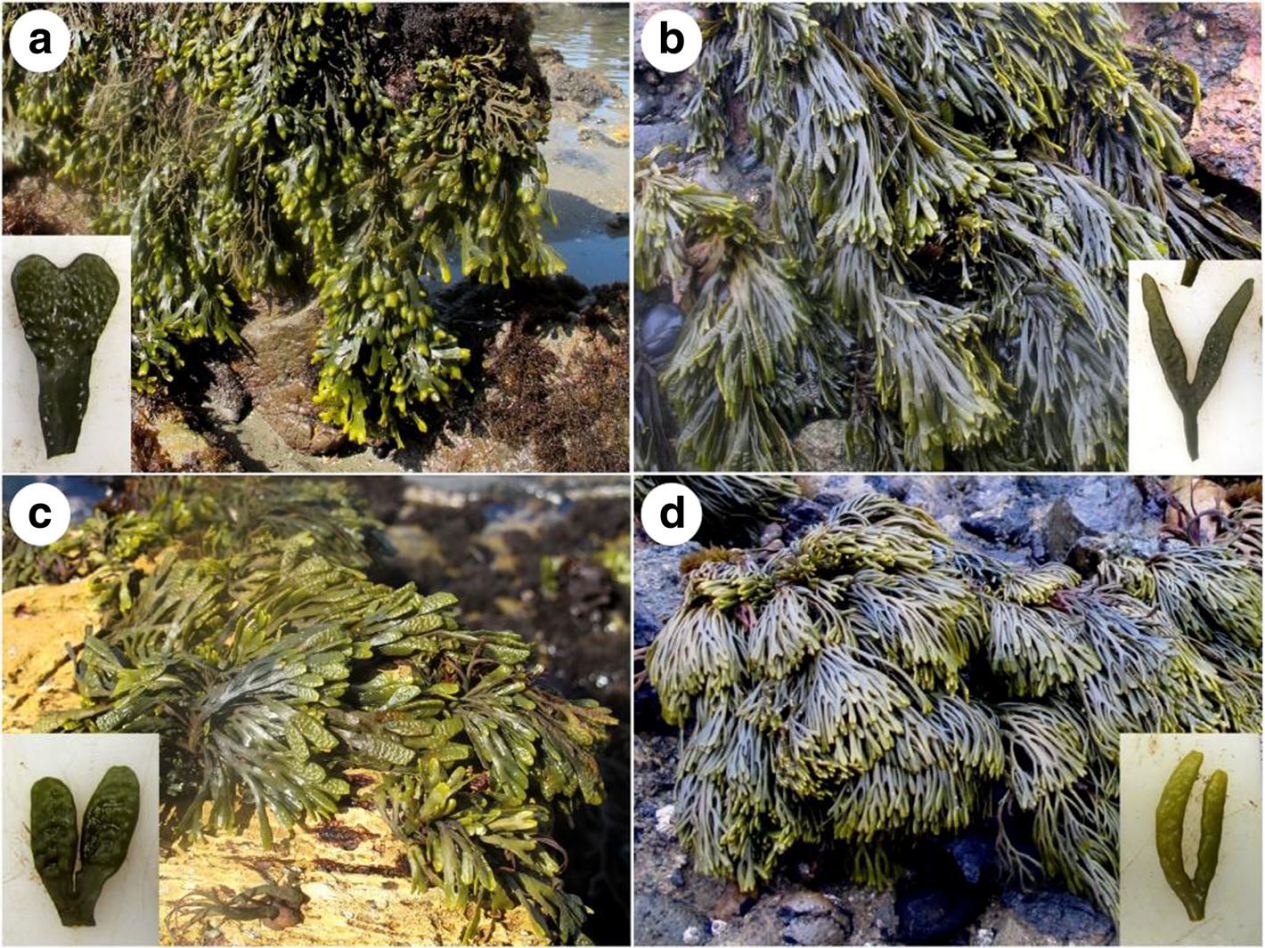
General morphology and shape of receptacles of *Pelvetiopsis* spp. **a** *Hesperophycus californicus* (=*Pelvetiopsis californica*), **b** *P. limitata*, **c** *P. hybrida*, and **d** *P. arborescens*

**Fig. 4:**
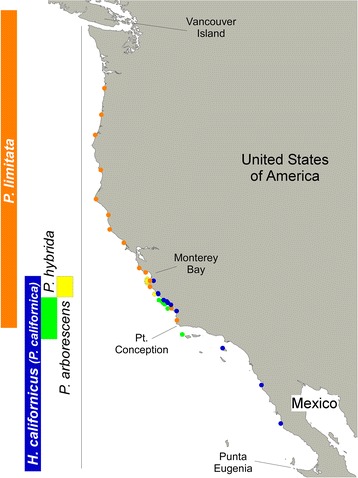
Inferred latitudinal distributions of *Pelvetiopsis* spp. Distributions based on the geographical origin of genetically-determined species samples (colour codes as in other figures), and also based in literature records. Note the overlapping ranges of species between Point Conception and Monterey Bay

*A posteriori*, all four genetic entities could be recognized based on their general morphology (Fig. 3). A combination of several characteristics, including maximum length, frond width, presence and distribution of cryptostomata in vegetative and reproductive parts, and shape of receptacles, was particularly helpful to confirm species identities in the field (Additional file 4). The large sampling effort of this study allowed us to establish the distribution range of each species (Fig. 4). The southernmost species, *H. californicus*, was genetically confirmed from Baja California to Monterey Bay whereas cold-temperate *P. limitata* was confirmed from Point Conception to N Oregon. *P. arborescens* and *P. hybrida* exhibited much more restricted (and apparently non-overlapping) distributions centered in the Monterey (Big Sur) and San Luis Obispo counties, respectively. The first was mostly found in the Carmel area, with the southernmost population (Pacific Valley) representing an isolated collection. *P. hybrida* was also found in remote San Miguel Island, and its distribution in other Channel Islands remains a possibility. No species were collected on the mainland between Mexico and Point Conception.

## Discussion

### Crypticism, allopolyploidy and biogeography of *Pelvetiopsis* spp.

Our analyses revealed multiple taxonomic, phylogenetic and genomic inconsistencies within *Hesperophycus-Pelvetiopsis*, notably the occurrence of 1) more genetic entities than the two initially recognized species, 2) species/gene conflicts, and 3) stable, genome-wide heterozygosity in two entities. Such conflicts often reflect incorrect taxonomy (e.g. cryptic species) or natural evolutionary processes such as incomplete lineage sorting and introgressive hybridization, among others [56]. Here, both types of factors were readily identified.

In contrast with *H. californicus*, genetic data clearly supported multiple entities within *P. limitata sensu lato*. One of these undoubtedly corresponds to the previously described *P. arborescens*, as confirmed by its characteristic morphology and occurrence at the type locality (Point Lobos, [39]). Another one, the newly recognized pseudo-cryptic *Pelvetiopsis hybrida*, was found to be genetically intermediate between *H. californicus* and *P. arborescens*, with all available evidence indicating an allopolyploid origin (see below). All four entities corresponded to genetically discontinuous clusters that were found *a posteriori* to further exhibit distinctive morphological characteristics. All combinations of species pairs (with the exception of *P. arborescens* with *P. hybrida*) were found locally co-occurring in at least a few sites between Point Conception and Monterey Bay (personal observations). Examples include *P. limitata*, *P. arborescens* and *H. californicus* all along the Monterey peninsula, *P. hybrida* and *H. californicus* throughout San Luis Obispo County, and *P. limitata* and *P. hybrida* in the same county around Hazards (Montaña de Oro State Park). Despite close proximity, local ongoing hybridization between species was never detected. Given their genetic and morphological integrity in sympatry, these four entities (*P. limitata, P. arborescens, P. hybrida,* and *H. californicus*) clearly correspond to good biological species.

Molecular analyses nevertheless revealed a key hybridization event in the evolutionary history of *Hesperophycus*-*Pelvetiopsis*. Specifically, *P. hybrida* (and to a lesser extent *P. limitata*) exhibited fixed heterozygosity in a high proportion of microsatellite loci, with allele states clearly indicating the contribution of *H. californicus* and *P. arborescens* genomes. Genomic heterozygosity in sexually reproducing organisms is not expected beyond F1 hybrids, because independent (Mendelian) segregation of chromosomes and successive generations of mating necessarily generate a proportion of homozygotes while pulling genotypic frequencies of populations towards Hardy-Weinberg equilibrium. In selfing species and/or with very restricted gametic dispersal, inbreeding would actually promote an excess of homozygotes, as observed here in *H. californicus* and *P. arborescens*, and in other fucoids sharing similar life-history and reproductive traits [57, 58]. *P. hybrida* cannot result from the recurrent formation of F1 hybrids, since currently it doesn’t overlap its distribution with *P. arborescens*, one of its inferred ancestors. A clonal hybrid swarm is also unlikely because *P. hybrida* produces fully functional sexual gametes (pers. observ.) and also because vegetative propagation by Fucaceae has never been reported in intertidal wave-swept habitats. Clonal lineages are present only in sheltered environments such as salt-marshes, estuarine mudflats and land-locked bays, and exhibit distinctive morphologies characterized by dwarf and curly habit, vegetative proliferation and lack (or nearly so) of reproductive structures and anchoring holdfasts ([59–62]). Attached, open-coast clones have only been reported from the atidal, brackish Baltic-Sea, where they result from the reattachment of breaking vegetative fragments [63]. This process requires motionless time (weeks) on a substrate to develop rhizoids, something that can happen in the Baltic subtidal where sea surface can be still for prolonged periods (for example, when it freezes, leaving the algae in still water underneath), but not in the wave-swept intertidal rocky-shores sites where *P. hybrida* occurs.

Under sexual reproduction (including selfing), only allopolyploidy can account for the generationally stable, genome-wide hybridity of *P. hybrida*. At least in selfing species, allopolyploidy is more likely to result from the self-fusion of unreduced gametes of F1 hybrids than via triploid bridges [64], although the actual pathways can only be guessed at. In any case, genome duplication may have been the key event restoring hybrid fertility, since *P. hybrida* originates from relatively divergent species. Despite the fixed heterozygosity of individuals, the very low number of individuals exhibiting three alleles (four were never observed) in at least one locus indicates that most homeolog loci actually occur, as expected in low-dispersal selfing species, in the homozygous state.

Organellar genomes are maternally inherited in brown seaweeds [65, 66]. Mitochondrial sequence data thus allow us to identify a northern female *H. californicus* and a male *P. arborescens* as the diploid ancestors of *P. hybrida.* The small mtDNA divergence and the (mainly) identical microsatellite allele states further argue for a relatively recent evolutionary origin. However, we show that mtDNA haplotypes are no longer shared between *P. hybrida* and *H. californicus*, and that the modern ranges of *P. hybrida* and *P. arborescens* do not overlap. Complete mtDNA lineage sorting and range shifts can occur on relatively short time-scales (single or few glacial cycles, [7, 29]), but here the results clearly indicate that hybridization was not extremely recent and is not ongoing presently. In *P. limitata*, microsatellite data was also compatible with multiple homeologue chromosomes, but allele states didn’t reveal any obvious relationship with any of its extant conspecifics. Ongoing karyotyping and sequencing of multiple nuclear loci will allow confirmation of the genealogical relationships and ploidies inferred here for *H. californicus*, *P. arborescens* and *P. hybrida*, and explore the hypothesis that *P. limitata* corresponds to a second, cryptic paleopolyploid species.

The hypothesis that *P. limitata* had also an allopolyploid origin is striking because currently this cold-temperate species shows the widest latitudinal span of all recognized species (*ca*. 14°), and is the only species occurring north of Monterey Bay. By comparison, *P. hybrida* showed a much more restricted range in mainland California and in San Miguel Island (Northern Channel Islands). Low-dispersal organisms might take some time to migrate and colonize new habitats. It can be hypothesized that this rather recent species is still in the process of equilibrating with its climatic niche (i.e., still expanding its range). Remarkably, its inferred paternal ancestor, *P. arborescens*, was only found in an even narrower, non-overlapping stretch of coastline spanning about 0.7° in latitude (<130 km). This degree of endemism is rare in temperate marine species, and in related fucoids is normally associated with newly emerged, extreme habitats (e.g. *Fucus radicans* in the brackish Baltic Sea, [45]) and/or past climates (e.g. *F. virsoides* in the Adriatic Sea, [67]). Within its small range, *P. arborescens* was always found in small scattered patches and only along a high intertidal fringe typically above all other intertidal fucoids (JN, EAS, GAP, pers. obs.). Notwithstanding its dynamic evolutionary history, currently this species may be better described as an extremely vulnerable climatic relict [22]. Finally, the most southern species, *H. californicus*, also exhibited a fairly wide latitudinal range, although south of Point Conception this was very discontinuous and mostly restricted to offshore islands (e.g. Northern and Southern Channel Islands) and prominent capes (e.g Punta Baja) [68, 69]. This species exhibited a marked phylogeographical break at Point Conception that should be further investigated.

The rich coastal biota of California, including its luxuriant seaweed flora, has been extensively surveyed in the past decades; the intertidal fucoid assemblages in particular are conspicuous and easily accessible for amateurs and trained phycologists, and are routinely included in coastal monitoring programs (e.g MARINe consortium). The discovery of a new *Pelvetiopsis* illustrates how much cryptic diversity may be hiding within more problematic groups and in more remote areas and depths. Remarkably, all *Pelvetiopsis* were present along the transitional coastline between Point Conception and Monterey Bay, either as relict (*P. arborescens*) or evolutionarily recent (*P. hybrida*) endemics, or, for more widespread species, overlapping the southernmost (cold-temperate *P. limitata*) and northernmost (warm-temperate *P. californica*) parts of their ranges. This modern hotspot of biodiversity, refugial persistence and evolutionary opportunity probably results from its relatively stable climatic history and high spatial habitat heterogeneity. The narrow endemic species nevertheless raise conservation concerns and deserve finer detail monitoring, particularly *P. arborescens*. Its restricted range, rarity and ecologically marginal vertical distribution make it particularly vulnerable to demographic stochasticity and to extreme El Niño events and ongoing climatic changes.

The identification of three phylogenetic clades comprising four species (one of hybrid origin) calls for a taxonomic revision of the complex (Table 2). Specifically, available data do not support the historical separation of *Hesperophycus* and *Pelvetiopsis,* and a new combination for *Hesperophycus californicus* (*Pelvetiopsis californica*) is therefore proposed. In addition to the formal description of the allotetraploid *P. hybrida*, the practical recognition of rare *P. arborescens* is also warranted.

**Table 2:** Taxonomic update of *Hesperophycus-Pelvetiopsis*

The original separation of the sister genera *Hesperophycus* and *Pelvetiopsis* was based on their evident morphological differences. However, the number of eggs per oogonium, the main phenetic character used to circumscribe genera in the Fucaceae, is identical (*n* = 1) in and unique to these genera. Genetic distances, as assessed with ITS (Serrão et al. [24]), multiple protein-coding loci (Cánovas et al. [40]) and mtDNA (this study, including *P. arborescens*), are invariably of the same order of magnitude as those separating the two main lineages within the sister genus *Fucus*. These genera produced a viable hybrid polyploid taxon, *P. hybrida*, which is genetically intermediate between them. In the absence of sufficient molecular or reproductive divergence to justify two distinct genera, *Hesperophycus* and *Pelvetiopsis* must be merged into a single, monophyletic genus containing the four extant species identified to date. These genera were typified on the same page of the same publication (Gardner [93]) to accommodate *Hesperophycus harveyanus* (previously *Fucus harveyanus* Decaisne 1864) and *Pelvetiopsis limitata* (previously *Pelvetia fastigiata* f. *limitata* Setchell 1905). Both names are legitimate and have equal priority. We therefore chose to keep *Pelvetiopsis* to minimize nomenclatural changes. In addition, the holotype of *Hesperophycus* was actually a specimen of *Fucus*, a situation that was corrected only in the early 1990’s (Silva [68]). We thus propose the extinction of the genus *Hesperophycus*, a new combination (*Pelvetiopsis californica*), and a new species (*Pelvetiopsis hybrida*).
*Pelvetiopsis* N.L. Gardner 1910
*Pelvetiopsis limitata* (Setchell) N.L. Gardner 1910 (type species)
*Pelvetiopsis arborescens* N.L. Gardner 1940
*Pelvetiopsis californica* (P.C. Silva) Neiva, J., Raimondi, P.T., Pearson, G.A., Serrão, E.A. comb. nov. *Basionym*: *Hesperophycus californicus* P.C. Silva 1990 (Taxon 39: 1-8)
*Pelvetiopsis hybrida* Neiva, J., Raimondi, P.T., Pearson, G.A., Serrão, E.A. sp. nov. *Diagnosis*: Perennial, general morphology and size similar to *Pelvetiopsis limitata*, but distinguished from it by the occurrence of cryptostomata on the abaxial side of young frond tips, and by blunt, usually not bifurcated, receptacles; distinguished from *Pelvetiopsis californica* by its smaller size (<20 cm) and by the absence of abundant cryptostomata on both sides of fronds (often arranged in lines) and on receptacles. Distinguished from *P. arborescens* by its larger size (>13 cm), wider fronds (2.5–5 mm width), and blunt receptacles. This is an allopolyploid species derived from *P. californica* and *P. arborescens*, as assessed by a cloned nuclear intron and multiple codominant microsatellite markers. *Holotype*: Otter Point, San Miguel Island, Santa Barbara County, California, USA. 17 February 2015; Kathy Ann Miller (12-I-2015); UC2050575. *Isotype*: UC2050576. *Paratypes*: UC2050573, UC2050574. *Etymology*: of hybrid origin. *Habitat*: Marine, intertidal. Growing on average above *P. californica* where they co-occur*. Distribution*: San Luis Obispo County and San Miguel Island, California.

### Originality of allopolyploid speciation

The recognition of two narrow-endemic, pseudo-cryptic species within *Pelvetiopsis* is certainly remarkable, but the allopolyploid nature of *P. hybrida* is truly unexpected and completely original both from a taxonomic (brown seaweeds) and ecological (intertidal) perspective. Variance in genome size estimates in seaweeds have been attributed to palaeo-polyploidization events [35, 70], including in Fucales [71], but direct genetic evidence for modern polyploids remains scarce [38, 72] and nearly non-existent in brown seaweeds (but see [60]. Autopolyploids are harder to detect genetically and may be underreported [73], but not allopolyploids exhibiting stable genomic hybridity (e.g. [74]). For instance, unlike *P. hybrida*, suspected *Macrocystis* x *Pelagophycus* allopolyploids could not be confirmed genetically based on this benchmark (revised in [75]). Surprisingly, positive correlates of plant polyploidy, including perenniality and ability to self, are common in many speciose genera of canopy-forming brown seaweeds (*Fucus*, *Sargassum*, *Cystoseira, Laminaria*, *Saccharina*), some of which also display some propensity to hybridize. For instance, in the closely related and well-studied genus *Fucus*, hybridization between related species has been frequent and ongoing but the primary evolutionary consequence has only been introgressive gene-flow [29–31].

Why is allopolyploidy featuring in recent *Pelvetiopsis* evolution while virtually unknown across its entire class? Globally, whole genome duplication appears to require relatively stringent conditions; hybridization for instance is taxonomically widespread and frequent [76], whereas polyploidy is common in plants [77] and a few vertebrate lineages [78] but infrequent in most other taxonomic groups [37, 79, 80]. Even in plants, high variation in the frequency of polyploidy implies different propensities for polyploidization, or at least polyploid establishment, in different lineages [81]. In related *Fucus*, chemotactic attraction of sperm by eggs is cross-specific [82], and the potential for gamete contact in mixed stands can be high (e.g., during synchronous joint spawning events, but see [83]). Yet, hybridization (leading to introgression) is only known between related species and is often asymmetric [29, 30]. For instance, natural hybrids normally involve male (sperm) *F. serratus* x female (eggs) *F. evanescens*, presumably because sperm allocation is much higher in dioecious vs hermaphrodite species [84]. Speciation of *P. hybrida* on the other hand involved the interaction of two divergent self-compatible hermaphroditic species. This is unlikely to be a coincidence. Allopolyploids seem to originate more frequently when crosses involve genomically divergent species ([85]; but see [86]) because more severe meiotic conflicts presumably prevent diploid hybrids from backcrossing/introgressing with parental species (or to self), and result in higher numbers of unreduced (diploid) gametes. This is consistent with inter-lineage allopolyploid speciation in *Pelvetiopsis* vs intra-lineage (most likely homoploid) introgression in sister *Fucus.* It does not really offer, however, a general explanation for why allopolyploidy is otherwise so rare among brown seaweeds.

It should be noted that *P. hybrida* was found co-occurring in sympatry with its ancestor *P. californica* (= *H. californicus*) throughout much of its distribution. Niche differentiation is acknowledged as a pre-requisite, or at least facilitator, allowing initial establishment and long-term survival of viable (but numerically rare) neopolyploids as stable species, by alleviating potential competition with longer-established diploid ancestors [87–90]. Both polyploidy and hybridization have saltation effects, and any adaptive phenotypic trait may be more readily conserved (and potentially reinforced) due to cytotype isolation. In any case, ecological differences need not be great. Floristic meta-analyses show that budding speciation (new species arising within the ranges of older, widespread persisting taxa) is common in plants, with sister species often differing in subtle habitat preferences conducive to fine-scale parapatry/allopatry [18]. The importance of fine-scale environmental heterogeneity and of reproductive traits (such as selfing or timing of reproduction) is also known to promote species divergence in the shallow marine realm. In sister *Fucus*, for instance, the integrity and vertical distribution of closely related species is maintained along strong vertical gradients by small differences in stress tolerance, even in the face of moderate gene-flow [14]. Similar patterns are also apparent in mixed *Pelvetiopsis* stands occurring between Point Conception and Monterey. The average vertical ranges of *P. hybrida* and *P. arborescens* were always above those of *H. californicus* and *P. limitata* where they co-occurred (unpublished data). Likewise, very local (e.g. boulder level) patterns of distribution suggest an important role for wave action in limiting their distributions. Whether these ecological differences are among the immediate effects of allopolyploidization is unclear, since they cannot be separated from other evolutionary processes (e.g. drift, selection) driving subsequent niche divergence [91].

Because of the very limited genomic differentiation of *P. hybrida* from its extant ancestors, this species is particularly suited to investigate how divergent parental genomes are transferred and functionally integrated in a new species and might contribute to adaptive diversification. The rocky intertidal zone provides one of the best natural laboratories for examining the relationships between abiotic constraints, biotic interactions, and ecological patterns in nature [92]. A few sites where different combinations of species coexist have already been identified, and field observations and experimental work may identify niche differences allowing coexistence at these local scales, including among species with partially shared genetic backgrounds.

## Conclusions

This study revealed a much more diverse and evolutionarily complex picture of NE Pacific *Pelvetiopsis* than previously anticipated. In addition to the confirmation of the Big Sur endemic *P. arborescens*, molecular data allowed for the first time the identification of a stable allopolyploid marine species (*P. hybrida*) with clearly discernable phylogenetic relationships (♀ *H. californicus* x ♂ *P. arborescens*), morphology, and geographical distribution. Ongoing analyses of nuclear sequence data support our inferences regarding *P. hybrida*, and are consistent with the hypothesis of *P. limitata* corresponding to a second, cryptic paleopolyploid. Allopolyploid speciation remains otherwise unknown in brown seaweeds, and until some ecological, biological or reproductive originality is identified, its unique occurrence within *Pelvetiopsis* will remain enigmatic. The diversity hotspot between Point Conception and Monterey Bay likely reflects its relatively stable climatic history and its regionally high habitat heterogeneity. This conspicuous seaweed genus from the well-studied Californian intertidal illustrates how much diversity (and evolutionary processes) potentially remains undiscovered in other (less studied) marine groups and regions.

## Additional files

Additional file 1: Genetic diversity within populations of *Pelvetiopsis* spp. Allelic richness (A, mean number of alleles per locus), Nei’s gene diversity (H_E_), observed heterozygosity (H_o_), and multi-locus inbreeding coefficient (F_IS_,) were estimated for each population of each inferred species. (DOCX 24 kb)
Additional file 2: Primer sequences and amplification details for all markers. (DOCX 20 kb)
Additional file 3: Microsatellite allele frequencies in populations of *Pelvetiopsis* spp. Loci are separated by vertical lines, with loci names on top and allele sizes (bp) on the bottom. The presence of an allele in a population is indicated by a circle with an area proportional to its frequency. Population codes as in Additional file 1. Horizontal lines separate the inferred species, where *P. californica* is a synonym of *Hesperophycus californicus*. Grey squares identify different allele sets co-occurring in each individual of *P. hybrida* and/or *P. limitata*. Dashed squares mark the allele sets where allele drop-out was detected. (DOCX 216 kb)
Additional file 4: Morphological characteristics useful for field discrimination of *Pelvetiopsis* spp. (DOCX 16 kb)

## References

[1] Gaston KJ (1996). Species-range-size distributions: Patterns, mechanisms and implications. Trends Ecol Evol.

[2] Vega GC, Wiens JJ (2012). Why are there so few fish in the sea?. Proc Biol Sci.

[3] Cowen RK, Sponaugle S (2009). Larval dispersal and marine population connectivity. Annu Rev Mar Sci.

[4] Neiva J, Pearson GA, Valero M, Serrão EA (2012). Drifting fronds and drifting alleles : the genetic architecture of the estuarine seaweed *Fucus ceranoides* L.J. Biogeogr.

[5] Hurtado LA, Mateos M, Santamaria CA (2010). Phylogeography of supralittoral rocky intertidal *Ligia* isopods in the pacific region from central California to central Mexico. PLoS One.

[6] Rastorgueff PA, Chevaldonné P, Arslan D, Verna C, Lejeusne C (2014). Cryptic habitats and cryptic diversity: Unexpected patterns of connectivity and phylogeographical breaks in a Mediterranean endemic marine cave mysid. Mol Ecol.

[7] McDevit DC, Saunders GW (2010). A DNA barcode examination of the Laminariaceae (Phaeophyceae) in Canada reveals novel biogeographical and evolutionary insights. Phycologia.

[8] Silberfeld T, Bittner L, Fernández-García C, Cruaud C, Rousseau F, de Reviers B (2013). Species diversity, phylogeny and large scale biogeographic patterns of the genus *Padina* (Phaeophyceae, Dictyotales). J Phycol.

[9] Vieira C, D’hondt S, De Clerck O, Payri CE (2014). Toward an inordinate fondness for stars, beetles and Lobophora? Species diversity of the genus *Lobophora* (Dictyotales, Phaeophyceae) in New Caledonia. J Phycol.

[10] Guillemin M-L, Contreras-Porcia L, Ramírez ME, Macaya EC, Contador CB, Woods H, et al. The bladed Bangiales (Rhodophyta) of the South Eastern Pacific: Molecular species delimitation reveals extensive diversity. Mol. Phylogenet. Evol. 2015;94:814–26. 10.1016/j.ympev.2015.09.027 26484942

[11] Tellier F, Tapia J, Faugeron S, Destombe C, Valero M (2011). The *Lessonia nigrescens* species complex (Laminariales, Phaeophyceae) shows strict parapatry and complete reproductive isolation in a secondary contact zone. J Phycol.

[12] Fraser CI, Spencer HG, Waters JM (2012). *Durvillaea poha* sp. nov. (Fucales, Phaeophyceae): a buoyant southern bull-kelp species endemic to New Zealand. Phycologia.

[13] Bergstrom L, Tatarenkov A, Johannesson K, Jonsson RB, Kautsky L (2005). Genetic and morphological identification of *Fucus radicans* sp. nov. (Fucales, Phaeophyceae) in the brackish Baltic Sea. J Phycol.

[14] Zardi GI, Nicastro KR, Canovas F, Costa JF, Serrão EA, Pearson GA (2011). Adaptive traits are maintained on steep selective gradients despite gene flow and hybridization in the intertidal zone. PLoS One.

[15] Cunha RL, Castilho R, Rüber L, Zardoya R (2005). Patterns of cladogenesis in the venomous marine gastropod genus *Conus* from the Cape Verde islands. Syst Biol.

[16] Meyer CP, Geller JB, Paulay G (2005). Fine scale endemism on coral reefs: Archipelagic differentiation in turbinid gastropods. Evolution.

[17] Payo DA, Leliaert F, Verbruggen H, D’hondt S, Calumpong HP, De Clerck O (2013). Extensive cryptic species diversity and fine-scale endemism in the marine red alga *Portieria* in the Philippines. Proc Biol Sci.

[18] Anacker BL, Strauss SY (2014). The geography and ecology of plant speciation: range overlap and niche divergence in sister species. Proc Biol Sci.

[19] Stuart AJ, Kosintsev PA, Higham TFG, Lister AM (2004). Pleistocene to Holocene extinction dynamics in giant deer and woolly mammoth. Nature.

[20] Dynesius M, Jansson R (2000). Evolutionary consequences of changes in species’ geographical distributions driven by Milankovitch climate oscillations. Proc Natl Acad Sci U S A.

[21] Bennett K, Provan J (2008). What do we mean by “refugia”?. Quat Sci Rev.

[22] Ohlemüller R, Anderson BJ, Araújo MB, Butchart SHM, Kudrna O, Ridgely RS (2008). The coincidence of climatic and species rarity: high risk to small-range species from climate change. Biol Lett.

[23] Lourenço CR, Zardi GI, McQuaid CD, Serrão EA, Pearson GA, Nicastro KR. Upwelling areas as climate change refugia for the distribution and genetic diversity of a marine macroalga. J. Biogeogr. 2016;43:1595–607.

[24] Serrão EA, Alice LA, Brawley SH (1999). Evolution of the Fucaceae (Phaeophyceae) inferred from nrDNA-ITS. J Phycol.

[25] Lane CE, Mayes C, Druehl LD, Saunders GW (2006). A multi-gene molecular investigation of the kelp (Laminariales, Phaeophyceae) supports substantial taxonomic re-organization. J Phycol.

[26] Silberfeld T, Leigh JW, Verbruggen H, Cruaud C, de Reviers B, Rousseau F (2010). A multi-locus time-calibrated phylogeny of the brown algae (Heterokonta, Ochrophyta, Phaeophyceae): Investigating the evolutionary nature of the “brown algal crown radiation”. Mol Phylogenet Evol.

[27] Arnold ML, Fogarty ND (2009). Reticulate evolution and marine organisms: The final frontier?. Int J Mol Sci.

[28] Willis BL, van Oppen MJH, Miller DJ, Vollmer SV, Ayre DJ (2006). The role of hybridization in the evolution of reef corals. Annu Rev Ecol Evol Syst.

[29] Neiva J, Pearson GA, Valero M, Serrão EA (2010). Surfing the wave on a borrowed board: range expansion and spread of introgressed organellar genomes in the seaweed *Fucus ceranoides* L. Mol Ecol.

[30] Coyer JA, Hoarau G, Stam WT, Olsen JL (2007). Hybridization and introgression in a mixed population of the intertidal seaweeds *Fucus evanescens* and *F. serratus*. J Evol Biol.

[31] Coyer JA, Hoarau G, Costa JF, Hogerdijk B, Serrão EA, Billard E (2011). Evolution and diversification within the intertidal brown macroalgae *Fucus spiralis*/*F. vesiculosus* species complex in the North Atlantic. Mol Phylogenet Evol.

[32] Mcfadden CS, Hutchinson MB (2004). Molecular evidence for the hybrid origin of species in the soft coral genus *Alcyonium* (Cnidaria: Anthozoa: Octocorallia). Mol Ecol.

[33] Vollmer SV, Palumbi SR (2002). Hybridization and the evolution of reef coral diversity. Science.

[34] Dehal P, Boore JL (2005). Two rounds of whole genome duplication in the ancestral vertebrate. PLoS Biol.

[35] Kapraun DF (2005). Nuclear DNA, content estimates in multicellular green, red and brown algae: phylogenetic considerations. Ann Bot.

[36] Ghiselli F, Milani L, Scali V, Passamonti M (2007). The *Leptynia hispanica* species complex (Insecta Phasmida): Polyploidy, parthenogenesis, hybridization and more. Mol Ecol.

[37] Albertin W, Marullo P (2012). Polyploidy in fungi: evolution after whole-genome duplication. Proc Biol Sci.

[38] Niwa K, Kobiyama A (2014). Speciation in the marine crop *Pyropia yezoensis* (Bangiales, Rhodophyta). J Phycol.

[39] Gardner N (1940). New species of Melanophyceae from the Pacific coast of North America. Univ Calif Publ Bot.

[40] Cánovas FG, Mota CF, Serrão EA, Pearson GA (2011). Driving south: a multi-gene phylogeny of the brown algal family Fucaceae reveals relationships and recent drivers of a marine radiation. BMC Evol Biol.

[41] Chapman ARO (1995). Functional ecology of fucoid algae: twenty-three years of progress. Phycologia.

[42] Pearson GA, Serrão EA (2006). Revisiting synchronous gamete release by fucoid algae in the intertidal zone: fertilization success and beyond?. Integr Comp Biol.

[43] Neiva J, Serrão EA, Assis J, Pearson GA, Coyer JA, Olsen JL, Hu Z-M, Fraser C (2016). Climate oscillations, range shifts and phylogeographic patterns of North Atlantic Fucaceae. Seaweed Phylogeography - Adapt. Evol. Seaweeds under Environ. Chang.

[44] Coyer JA, Hoarau G, Oudot-Le Secq M-P, Stam WT, Olsen JL (2006). A mtDNA-based phylogeny of the brown algal genus *Fucus* (Heterokontophyta; Phaeophyta). Mol Phylogenet Evol.

[45] Pereyra RT, Bergström L, Kautsky L, Johannesson K (2009). Rapid speciation in a newly opened postglacial marine environment, the Baltic Sea. BMC Evol Biol.

[46] Neiva J, Assis J, Fernandes F, Pearson GA, Serrão EA (2014). Species distribution models and mitochondrial DNA phylogeography suggest an extensive biogeographical shift in the high-intertidal seaweed *Pelvetia canaliculata*. J Biogeogr.

[47] Faircloth BC (2008). MSATCOMMANDER: Detection of microsatellite repeat arrays and automated, locus-specific primer design. Mol Ecol Resour.

[48] Toonen RJ, Hughes S (2001). Increased throughput for fragment analysis on an ABI Prism® 377 automated sequencer using a membrane comb and STRand software. Biotechniques.

[49] Bandelt HJ, Forster P, Röhl A (1999). Median-joining networks for inferring intraspecific phylogenies. Mol Biol Evol.

[50] Darriba D, Taboada GL, Doallo R, Posada D (2012). jModelTest 2: more models, new heuristics and parallel computing. Nat Methods.

[51] Ronquist F, Teslenko M, Van Der Mark P, Ayres DL, Darling A, Höhna S (2012). Mrbayes 3.2: Efficient bayesian phylogenetic inference and model choice across a large model space. Syst Biol.

[52] Guindon S, Dufayard JF, Lefort V, Anisimova M, Hordijk W, Gascuel O (2010). New algorithms and methods to estimate Maximum-Likelihood phylogenies: Assessing the performance of PhyML 3.0. Syst Biol.

[53] Belkhir K, Borsa P, Chikhi L, Raufaste N, Bonhomme F (1996). GENETIX 4.05, logiciel sous Windows TM pour la génétique des populations.

[54] Pritchard JK, Stephens M, Donnelly P (2000). Inference of population structure using multilocus genotype data. Genetics.

[55] Falush D, Stephens M, Pritchard JK (2003). Inference of population structure using multilocus genotype data: linked loci and correlated allele frequencies. Genetics.

[56] Funk DJ, Omland KE (2003). Species-level paraphyly and polyphyly: Frequency, causes, and consequences, with insights from animal mitochondrial DNA. Annu Rev Ecol Evol Syst.

[57] Perrin C, Daguin C, Van De Vliet M, Engel CR, Pearson GA, Serrão EA (2007). Implications of mating system for genetic diversity of sister algal species: *Fucus spiralis* and *Fucus vesiculosus* (Heterokontophyta, Phaeophyceae). Eur J Phycol.

[58] Neiva J, Assis J, Coelho NC, Fernandes F, Pearson GA, Serrão EA (2015). Genes left behind: Climate change threatens cryptic genetic diversity in the canopy-forming seaweed *Bifurcaria bifurcata*. PLoS One.

[59] Brinkhuis BH (1976). The ecology of temperate salt-marsh fucoids. I. Occurrence and distribution of *Ascophyllum nodosum* ecads. Mar Biol.

[60] Coyer JA, Hoarau G, Pearson GA, Serrão EA, Stam WT, Olsen JL (2006). Convergent adaptation to a marginal habitat by homoploid hybrids and polyploid ecads in the seaweed genus *Fucus*. Biol Lett.

[61] Mathieson AC, Dawes CJ, Wallace AL, Klein AS (2006). Distribution, morphology, and genetic affinities of dwarf embedded *Fucus* populations from the Northwest Atlantic Ocean. Bot Mar.

[62] Neiva J, Hansen GI, Pearson GA, Van De Vliet MS, Maggs CA, Serrão EA (2012). *Fucus cottonii* (Fucales, Phaeophyceae) is not a single genetic entity but a convergent salt-marsh morphotype with multiple independent origins. Eur J Phycol.

[63] Tatarenkov A, Bergström L, Jönsson RB, Serrão EA, Kautsky L, Johannesson K (2005). Intriguing asexual life in marginal populations of the brown seaweed *Fucus vesiculosus*. Mol Ecol.

[64] Ramsey J, Schemske DW (1998). Pathways, mechanisms, and rates of polyploid formation in flowering plants. Annu Rev Ecol Syst.

[65] Coyer JA, Peters AF, Hoarau G, Stam WT, Olsen JL (2002). Inheritance patterns of ITS1, chloroplasts and mitochondria in artificial hybrids of the seaweeds *Fucus serratus* and *F. evanescens* (Phaeophyceae). Eur J Phycol.

[66] Motomura T, Nagasato C, Kimura K (2010). Cytoplasmic inheritance of organelles in brown algae. J Plant Res.

[67] Orlando-Bonaca M, Mannoni P-A, Poloniato D, Falace A (2013). Assessment of *Fucus virsoides* distribution in the Gulf of Trieste (Adriatic Sea) and its relation to environmental variables. Bot Mar.

[68] Silva PC (1990). Hesperophycus Setchell & Gardner, nom. cons. prop., a problematic name applied to a distinctive genus of Fucaceae (Phaeophyceae). Taxon.

[69] Aguilar-Rosas R, Aguilar-Rosas LE, Mateo-Cid LE, Mendoza-González AC, Krauss-Cosio H (2002). *Hesperophycus* y *Silvetia* representantes de la familia Fucaceae (Fucales, Phaeophyta) en la costa del Pacífico de México. Hidrobiológica.

[70] Phillips N, Kapraun DF, Gómez Garreta A, Ribera Siguan MA, Rull JL, Salvador Soler N (2011). Estimates of nuclear DNA content in 98 species of brown algae (Phaeophyta). AoB Plants.

[71] Garreta AG, Siguan MAR, Soler NS, Lluch JR, Kapraun DF (2010). Fucales (Phaeophyceae) from Spain characterized by large-scale discontinuous nuclear DNA contents consistent with ancestral cryptopolyploidy. Phycologia.

[72] Niwa K, Sakamoto T (2010). Allopolyploidy in natural and cultivated populations of *Porphyra* (Bangiales, Rhodophyta)1. J Phycol.

[73] Barker MS, Arrigo N, Baniaga AE, Li Z, Levin DA. On the relative abundance of autopolyploids and allopolyploids. New Phytol. 2016;210: 391–98. 10.1111/nph.13698 26439879

[74] Estep MC, McKain MR, Vela Diaz D, Zhong J, Hodge JG, Hodkinson TR (2014). Allopolyploidy, diversification, and the Miocene grassland expansion. Proc Natl Acad Sci U S A.

[75] Druehl LD, Collins JD, Lane CE, Saunders GW (2005). An evaluation of methods used to assess intergeneric hybridization in kelp using Pacific Laminariales (Phaeophyceae). J Phycol.

[76] Mallet J (2007). Hybrid speciation. Nature.

[77] Soltis DE, Albert VA, Leebens-Mack J, Bell CD, Paterson AH, Zheng C (2009). Polyploidy and angiosperm diversification. Am J Bot.

[78] Mable BK, Alexandrou MA, Taylor MI (2011). Genome duplication in amphibians and fish: an extended synthesis. J Zool.

[79] Koester J, Swalwell J, von Dassow P, Armbrust E (2010). Genome size differentiates co-occurring populations of the planktonic diatom *Ditylum brightwellii* (Bacillariophyta). BMC Evol Biol.

[80] Varela-Álvarez E, Gómez Garreta A, Rull Lluch J, Salvador Soler N, Serrão EA, Siguán MAR (2012). Mediterranean species of *Caulerpa* are polyploid with smaller genomes in the invasive ones. PLoS One.

[81] Weiss-Schneeweiss H, Emadzade K, Jang T-S, Schneeweiss GMM (2013). Evolutionary consequences, constraints and potential of polyploidy in plants. Cytogenet Genome Res.

[82] Müller DG, Seferiadis K (1977). Specificity of sexual chemotaxis in *Fucus serratus* and *Fucus vesiculosus* (Phaeophyceae). Z Pflanzenphysiol.

[83] Monteiro CA, Paulino C, Jacinto R, Serrão EA, Pearson GA (2016). Temporal windows of reproductive opportunity reinforce species barriers in a marine broadcast spawning assemblage. Sci Rep.

[84] Vernet P, Harper JL (1980). The costs of sex in seaweeds. Biol J Linn Soc.

[85] Paun O, Forest F, Fay MF, Chase MW (2009). Hybrid speciation in angiosperms: Parental divergence drives ploidy. New Phytol.

[86] Buggs RJ, Soltis PS, Soltis DE (2009). Does hybridization between divergent progenitors drive whole-genome duplication?. Mol Ecol.

[87] Ainouche ML, Baumel A, Salmon A, Yannic G (2003). Hybridization, polyploidy and speciation in *Spartina* (Poaceae). New Phytol.

[88] Brochmann C, Brysting AK, Alsos IG, Borgen L, Grundt HH (2004). Polyploidy in arctic plants. Biol J Linn Soc.

[89] Mráz P, Garcia-Jacas N, Gex-Fabry E, Susanna A, Barres L, Müller-Schärer H (2012). Allopolyploid origin of highly invasive *Centaurea stoebe* s.l. (Asteraceae). Mol Phylogenet Evol.

[90] te Beest M, Le Roux JJ, Richardson DM, Brysting AK, Suda J, Kubesová M (2012). The more the better? The role of polyploidy in facilitating plant invasions. Ann Bot.

[91] Maherali H, Walden AE, Husband BC (2009). Genome duplication and the evolution of physiological responses to water stress. New Phytol.

[92] Helmuth B, Mieszkowska N, Moore P, Hawkins SJ (2006). Living on the edge of two changing worlds: Forecasting the responses of rocky intertidal ecosystems to climate change. Annu Rev Ecol Evol Syst.

[93] Gardner NL (1910). Variations in nuclear extrusion among the Fucaceae. Univ. Calif. Publ. Bot..

