# Supplementary material for: Cryptic diversity, geographical endemism and allopolyploidy in NE Pacific seaweeds
Source: BMC Evol Biol. 2017 Jan 23;17:30. doi: 10.1186/s12862-017-0878-2 (PMC5260064; doi:10.1186/s12862-017-0878-2)
Supplement: Additional file 1: — Genetic diversity within populations of Pelvetiopsis spp. Allelic richness (A, mean number of alleles per locus), Nei’s gene diversity (HE), observed heterozygosity (Ho), and multi-locus inbreeding coefficient (FIS,) were estimated for each population of each inferred species. (DOCX 24 kb) [file 12862_2017_878_MOESM1_ESM.docx]

Additional file 1. Genetic diversity within populations of *Pelvetiopsis* spp.

Allelic richness (A, mean number of alleles per locus), Nei’s gene diversity (H_E_), observed heterozygosity (H_o_), and multi-locus inbreeding coefficient (F_IS_,) were estimated for each population of each inferred species.

| **Sampled**  **as** | **New name** | **Population** | **Code** | **Coordinates**  **(decimal degrees)** | | **Microsatellites**  **(n_pop_≈16)** | | | |
| --- | --- | --- | --- | --- | --- | --- | --- | --- | --- |
|  |  |  |  | **Lat.** | **Long.** | ***A*** | ***H*_E_** | ***H*_O_** | ***F*_IS_** |
| ***Pelvetiopsis limitata sensu lato*** | *Pelvetiosis*  *limitata* | Stairs | STA | 34.7304 | -120.6151 | 1.46 | 0.238 | 0.462 | -1.000 ^<^ |
|  |  | Hazards | HAZ | 35.2896 | -120.8833 | 1.69 | 0.261 | 0.462 | -0.818 ^<^ |
|  |  | Andrew Molera | AMO | 36.2806 | -121.8632 | 1.54 | 0.241 | 0.457 | -0.959 ^<^ |
|  |  | Stillwater Cove | SWC | 36.5609 | -121.9404 | 1.46 | 0.238 | 0.462 | -1.000 ^<^ |
|  |  | Terrace Point | TPO | 36.9485 | -122.0648 | 1.69 | 0.255 | 0.440 | -0.770 ^<^ |
|  |  | Franklin Point | FPO | 37.1495 | -122.3610 | 1.77 | 0.261 | 0.467 | -0.838 ^<^ |
|  |  | Bodega Bay | BOD | 38.3182 | -123.0736 | 1.62 | 0.246 | 0.467 | -0.957 ^<^ |
|  |  | Stornetta | STO | 38.9379 | -123.7290 | 1.62 | 0.249 | 0.462 | -0.909 ^<^ |
|  |  | Kibesillah Hill | KIH | 39.6041 | -123.7888 | 1.69 | 0.258 | 0.462 | -0.840 ^<^ |
|  |  | Cape Mendoncino | CAM | 40.3412 | -124.3630 | 1.54 | 0.243 | 0.462 | -0.962 ^<^ |
|  |  | Enderts Beach | EBE | 41.6900 | -124.1426 | 1.46 | 0.239 | 0.462 | -1.000 ^<^ |
|  |  | Cape Arago | CAA | 43.3089 | -124.4005 | 1.77 | 0.295 | 0.476 | -0.646 ^<^ |
|  |  | Bob Creek | BOB | 44.2446 | -124.1143 | 1.54 | 0.243 | 0.462 | -0.962 ^<^ |
|  |  | Cape Meares | CPM | 45.4718 | -123.9722 | 1.46 | 0.238 | 0.462 | -1.000 ^<^ |
|  | *Pelvetiopsis arborescens* | Pacific Valley | PAV | 35.9471 | -121.4805 | 1.10 | 0.012 | 0.013 | -0.034 |
|  |  | Garrapata | GAR | 36.4689 | -121.9343 | 1.30 | 0.048 | 0.000 | 1.000 ^>^ |
|  |  | Point Lobos | PLO | 36.5132 | -121.9447 | 1.10 | 0.048 | 0.013 | 0.748 ^>^ |
|  |  | Pescadero | PES | 36.5611 | -121.9544 | 1.10 | 0.039 | 0.000 | 1.000 ^>^ |
|  |  | Point Pinos | PPI | 36.6380 | -121.9376 | 1.00 | - | - | - |
|  | *Pelvetiopsis*  *hybrida* | San Miguel Isl. | SMI | 34.0761 | -120.3678 | 2.00 | 0.411 | 0.725 | -0.809 ^<^ |
|  |  | Hazards | HAZ | 35.2896 | -120.8833 | 1.80 | 0.362 | 0.666 | -0.891 ^<^ |
|  |  | Harmony Headlands | HAR | 35.4746 | -121.0170 | 1.80 | 0.364 | 0.681 | -0.926 ^<^ |
|  |  | Rancho Marino | RMR | 35.5403 | -121.0928 | 2.10 | 0.434 | 0.731 | -0.725 ^<^ |
|  |  | San Simeon | SSI | 35.6044 | -121.1423 | 1.80 | 0.375 | 0.682 | -0.873 ^<^ |
|  |  | Piedras Blancas | PBL | 35.6657 | -121.2865 | 1.90 | 0.394 | 0.699 | -0.821 ^<^ |
| ***Hesperophycus* *californicus*** | *Pelvetiopsis californica* | Punta Baja | PBA | 29.9550 | -115.8062 | 1.11 | 0.035 | 0 | 1.000 ^>^ |
|  |  | Ensenada | ENS | 31.7344 | -116.7005 | 2.00 | 0.168 | 0.056 | 0.677 ^>^ |
|  |  | Santa Catalina Isl. | CTH | 33.4444 | -118.4989 | 1.44 | 0.182 | 0.083 | 0.549 ^>^ |
|  |  | Shell Beach | SBE | 35.1595 | -120.6861 | 1.00 | - | - | - |
|  |  | Cayucos | CAY | 35.4475 | -120.9501 | 1.11 | 0.057 | 0.021 | 0.643 ^>^ |
|  |  | Rancho Marino | RMR | 35.5403 | -121.0928 | 1.11 | 0.025 | 0.000 | 1.000 ^>^ |
|  |  | San Simeon | SSI | 35.6044 | -121.1423 | 1.00 | - | - | - |
|  |  | Piedras Blancas | PBL | 35.6657 | -121.2865 | 1.00 | - | - | - |
|  |  | Pacific Valley | PAV | 35.9471 | -121.4805 | 1.22 | 0.070 | 0.000 | 1.000 ^>^ |
|  |  | Mill Creek | MCK | 35.9798 | -121.4905 | 1.00 | - | - | - |
|  |  | Stillwater Cove | SWC | 36.5609 | -121.9404 | 1.00 | - | - | - |

^<^ significant homozygote deficit; ^>^ significant homozygote excess
