# Supplementary material for: Cryptic diversity, geographical endemism and allopolyploidy in NE Pacific seaweeds
Source: BMC Evol Biol. 2017 Jan 23;17:30. doi: 10.1186/s12862-017-0878-2 (PMC5260064; doi:10.1186/s12862-017-0878-2)
Supplement: Additional file 2: — Primer sequences and amplification details for all markers. (DOCX 20 kb) [file 12862_2017_878_MOESM2_ESM.docx]

### Additional file 2. Primer sequences and amplification details for all markers.

|  | **Locus**  **(Accession numbers)** | ***Motif*** | ***Primers***  ***5’ 🡪 3’*** | ***Mg***  ***(mM)*** | ***T_a_***  ***(*˚*C)*** | ***t_e_***  ***(s)*** | ***Cycles***  ***(n)*** |
| --- | --- | --- | --- | --- | --- | --- | --- |
| **Sequence markers** | mtIGS – Universal |  | F: TGGGTAGTTTGACTGGGGCGGT | 2.0 | 62 | 90 | 30 |
|  |  |  | R: ACGGTTCCAATACCCACACCTGC |  |  |  |  |
|  | mtIGS – *Pelvetiopsis* |  | F: GGGAGGTGCAAGAGCTGCAA | 2.0 | 58 | 60 | 30 |
|  |  |  | R: TCGAACTCCCGTCTTCGTGCT |  |  |  |  |
| **Microsatellite**  **markers** | Pl25 (KU872191) | (AT)_12_ | F: TGGGTCCAAATAACGTGCTA | 2.0 | 65* | 40 | 35 |
|  |  |  | R: CACCCTTGGCTTGACTGTATAA |  |  |  |  |
|  | Pl27 (KU872192) | (CA)13 | F:CACAGCATCTCCAAGCCC | 2.0 | 66* | 40 | 35 |
|  |  |  | R:CCGCGTGTGTATGTTTGTGTAT |  |  |  |  |
|  | Pl29 (KU872193) | (TA)10 | F:TTATACAAAACCCTCGCAAACC | 2.0 | 65* | 40 | 35 |
|  |  |  | R:ACAGGAGGACGAGCACTAAAAT |  |  |  |  |
|  | Pl32 (KU872194) | (CAA)7 | F:GGGCGTCAATATAAGAAGCGT | 2.0 | 65* | 40 | 35 |
|  |  |  | R:ATTTTCTGTCCTGCTGGGCT |  |  |  |  |
|  | Pl36 (KU872195) | (GTT)6 | F:GCTTATCATTTCCCACATCCAT | 2.5 | 60 | 40 | 35 |
|  |  |  | R:TCACCGTAGTTTCTTCAGTTGC |  |  |  |  |
|  | Pl39 (KU872196) | (GCT)9 | F:CGAAACAAGAAAAGGAGAAGGA | 2.0 | 60 | 40 | 35 |
|  |  |  | R:CCCCAATAATCTAGGCCACTCT |  |  |  |  |
|  | Pl310 (KU872197) | (TGT)8 | F:TTCTCCACTTGCTGTTCATTGT | 2.5 | 60 | 40 | 35 |
|  |  |  | R:GTGATGGCGTTCCATTTTACTT |  |  |  |  |
|  | Pl311 (KU872198) | (TTG)12 | F:ACTGCCCTTCGTCTACTTTCAG | 2.0 | 58 | 40 | 35 |
|  |  |  | R:TTTTATCGCTCTGTTGACCCTT |  |  |  |  |
|  | Pl41 (KU872199) | (CATA)10 | F:CAACTTTACCCGACTGATCCAT | 2.0 | 62 | 40 | 35 |
|  |  |  | R:CAAACGAAACATACTCATCCCA |  |  |  |  |
|  | Pl51 (KU872200) | (CCAAA)6 | F:GGAGGAGACGAATGAAACAAAT | 2.0 | 65* | 40 | 35 |
|  |  |  | R:ACGTGGAGATACGAGTTTGGAT |  |  |  |  |
|  | Pl52 (KU872201) | (GGTAT)6 | F:CGAGAATGAACAGCAACATCC | 2.0 | 65* | 40 | 35 |
|  |  |  | R:CTTATGGGAAGAAGACTCACGC |  |  |  |  |
|  | Pl53 (KU872202) | (GTATA)8 | F:AAGAGATGCTTACCGTCGTGA | 2.0 | 61 | 40 | 35 |
|  |  |  | R:AGGTGCGTTCAGATGTTTCTTT |  |  |  |  |
|  | Pl54 (KU872203) | (TACTA)7 | F:TTTGACACTTTCTCTCCTGCTG | 2.0 | 65* | 40 | 35 |
|  |  |  | R:TGTTTGTTATCGGGTTGAGTTG |  |  |  |  |

Each polymerase chain reaction (PCR) was performed in 20 (sequence markers) or 15 (microsatellite loci) μL total volume. These contained 1× taq buffer, 2.0-2.5 mM MgCl_2_, 125 μM each dNTP, 0.5 μM each primer (except labelled forward primers of microsatellites, of which only 0.2 μM were used), 1U GoTaq^®^ Flexi DNA Polymerase (Promega), and 5 μL of diluted (1:100) DNA template. PCRs involved an initial denaturation step (94 ˚C, 5 min), followed by n cycles of 3 steps consisting of 1) a denaturation step of 94˚C for 30s, 2) an annealing step at a primer-specific temperature T_a_ for 30s, and 3) an extension step of 72˚C during t_e_ seconds, and ended with a final extension step (72 ˚C, 20min). * In touchdown PCRs, initial T_a_ was reduced by 0.5 **˚** every new cycle during 10 cycles, followed by 25 cycles using T_a_ - 5 **˚.**
