# Supplementary material for: Cryptic diversity, geographical endemism and allopolyploidy in NE Pacific seaweeds
Source: BMC Evol Biol. 2017 Jan 23;17:30. doi: 10.1186/s12862-017-0878-2 (PMC5260064; doi:10.1186/s12862-017-0878-2)
Supplement: Additional file 3: — Microsatellite allele frequencies in populations of Pelvetiopsis spp. Loci are separated by vertical lines, with loci names on top and allele sizes (bp) on the bottom. The presence of an allele in a population is indicated by a circle with an area proportional to its frequency. Population codes as in Additional file 1. Horizontal lines separate the inferred species, where P. californica is a synonym of Hesperophycus californicus. Grey squares identify different allele sets co-occurring in each individual of P. hybrida and/or P. limitata. Dashed squares mark the allele sets where allele drop-out was detected. (DOCX 216 kb) [file 12862_2017_878_MOESM3_ESM.docx]

###
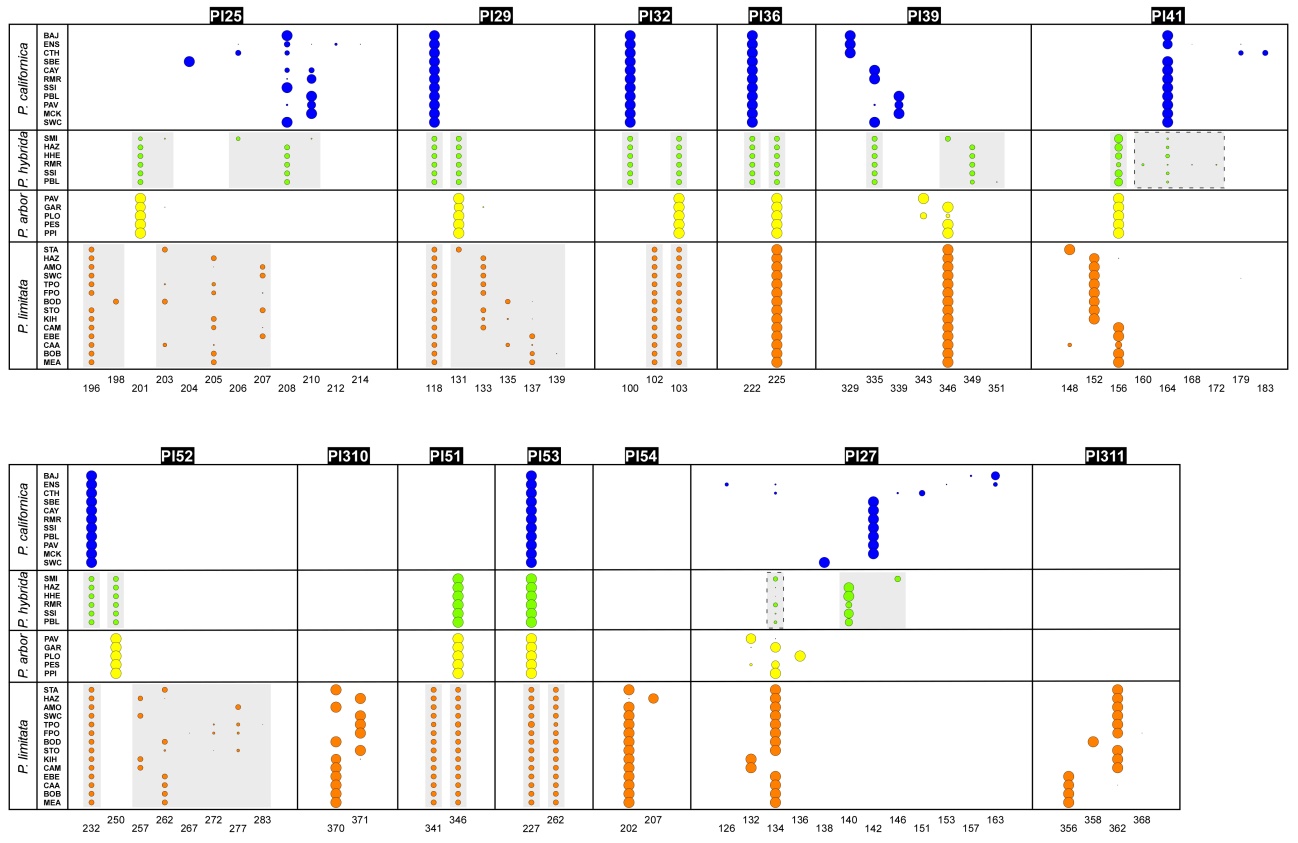


### Additional file 3. Microsatellite allele frequencies in populations of *Pelvetiopsis* spp.

Loci are separated by vertical lines, with loci names on top and allele sizes (bp) on the bottom. The presence of an allele in a population is indicated by a circle with an area proportional to its frequency. Population codes as in Additional file 1. Horizontal lines separate the inferred species, where *P. californica* is a synonym of *Hesperophycus californicus*. Grey squares identify different allele sets co-occurring in each individual of *P. hybrida* and/or *P. limitata*. Dashed squares mark the allele sets where allele drop-out was detected.
