# Supplementary material for: Cryptic diversity, geographical endemism and allopolyploidy in NE Pacific seaweeds
Source: BMC Evol Biol. 2017 Jan 23;17:30. doi: 10.1186/s12862-017-0878-2 (PMC5260064; doi:10.1186/s12862-017-0878-2)
Supplement: Additional file 4: — Morphological characteristics useful for field discrimination of Pelvetiopsis spp. (DOCX 16 kb) [file 12862_2017_878_MOESM4_ESM.docx]

**Additional file 4. Morphological characteristics useful for field discrimination of *Pelvetiopsis* spp.**

|  | **Maximum**  **length (cm)** | **Frond width***  **(mm)** | **Cryptostomata** | | **Shape of**  **receptacles** |
| --- | --- | --- | --- | --- | --- |
|  |  |  | fronds | receptacles |  |
| *P. californica*  *(= Hesperophycs californicus)* | 40 | 5.5 – 8 | both sides throughout,  generally arranged in two parallel rows | abundant throughout | oval to spatulate, flat to swollen, usually simple |
| *Pelvetiopsis*  *hybrida* | 20 | 2.5 – 5 | abaxial side only, in two parallel rows, or a few scattered, or absent | absent | spatulate, flat, usually simple |
| *Pelvetiopsis arborescens* | 13 | 1 – 2 | apical, abaxial side | absent | elongated, pointed, simple or bifurcated |
| *Pelvetiopsis limitata* | 20 | 2.5 – 5 | absent | absent | elongated, pointed, often bifurcated |

*Measured immediately below the receptacles
